# Supplementary figures and images for: Sex-Biased Expression of Pharmacogenes across Human Tissues
Source: Biomolecules. 2021 Aug 13;11(8):1206. doi: 10.3390/biom11081206 (PMC8393247; doi:10.3390/biom11081206)

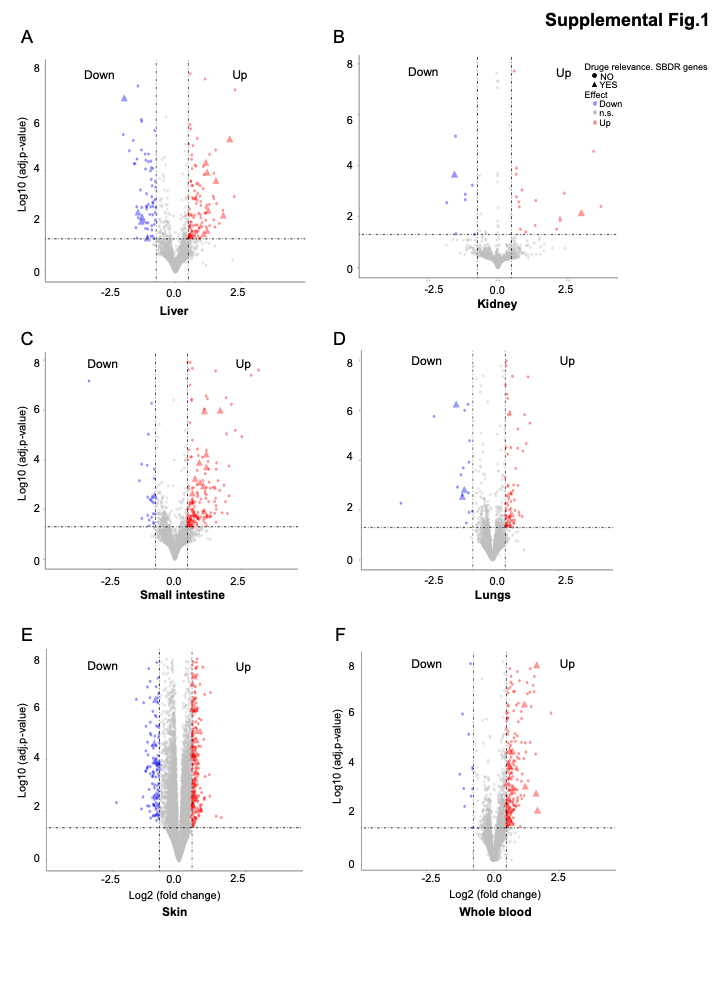

Supplement: Supplementary file 1 [file biomolecules-11-01206-s001.zip › Suppl Figure s1.png]
